# Detection of silent infection of severe acute respiratory syndrome coronavirus 2 by serological tests

**DOI:** 10.1371/journal.pone.0267566

**Published:** 2022-05-20

**Authors:** Masashi Nishimura, Satoshi Sugawa, Shinichiro Ota, Etsuko Suematsu, Masahiro Shinoda, Masaharu Shinkai

**Affiliations:** 1 Department of Respiratory Medicine, Tokyo Shinagawa Hospital, Shinagawa-Ku, Tokyo, Japan; 2 Core Diagnostics, Abbott Japan LLC, Minato-Ku, Tokyo, Japan; 3 Department of physiology, Showa University School of Medicine, Shinagawa-Ku, Tokyo, Japan; 4 Department of Clinical Laboratory Medicine, Tokyo Shinagawa Hospital, Shinagawa-Ku, Tokyo, Japan; University of Hail, SAUDI ARABIA

## Abstract

**Background:**

To control COVID-19 pandemic is of critical importance to the global public health. To capture the prevalence in an accurate and timely manner and to understand the mode of nosocomial infection are essential for its preventive measure.

**Methods:**

We recruited 685 healthcare workers (HCW’s) at Tokyo Shinagawa Hospital prior to the vaccination with COVID-19 vaccine. Sera of the subjects were tested by assays for the titer of IgG against S protein’s receptor binding domain (IgG (RBD)) or IgG against nucleocapsid protein (IgG (N)) of SARS-CoV-2. Together with PCR data, the positive rates by these methods were evaluated.

**Results:**

Overall positive rates among HCW’s by PCR, IgG (RBD), IgG (N) with a cut-off of 1.4 S/C (IgG (N)_1.4_), and IgG (N) with a cut-off of 0.2 S/C (IgG (N)_0.2_) were 3.5%, 9.5%, 6.1%, and 27.7%, respectively. Positive rates of HCW’s working in COVID-19 ward were significantly higher than those of HCW’s working in non-COVID-19 ward by all the four methods. Concordances of IgG (RBD), IgG (N)_1.4_, and IgG (N)_0.2_ against PCR were 97.1%, 71.4%, and 88.6%, respectively. By subtracting the positive rates of PCR from that of IgG (RBD), the rate of overall silent infection and that of HCW’s in COVID-19 ward were estimated to be 6.0% and 21.1%, respectively.

**Conclusions:**

For the prevention of nosocomial infection of SARS-CoV-2, identification of silent infection is essential. For the detection of ongoing infection, periodical screening with IgG (RBD) in addition to PCR would be an effective measure. For the surveillance of morbidity in the population, on the other hand, IgG (N)_0.2_ could be the most reliable indicator among the three serological tests.

## Background

COVID-19, a disease caused by severe acute respiratory syndrome coronavirus 2 (SARS-CoV-2) is reported to have emerged in December 2019 and caused a worldwide pandemic [1]. As of March 2022, worldwide cumulative number of cases and that of deaths by COVID-19 are 453 million and 6.0 million, respectively [2]. Vaccination against SARS-CoV-2 is hailed as one of the most effective measures to prevent the spread of the infection. Since the end of 2020, the vaccination has been promoted vigorously all over the world and the total vaccine doses including the second and third doses have reached to 10.7 billion as of March 2022 [2]. The effectiveness of the vaccines, however, may be compromised due to declining antibody titer and emergence of the viral variant, such as delta or omicron strain of SARS-CoV-2 [3, 4]. Therefore, even after the expansion of the vaccination to the whole population, we may still be under the circumstance in which we see the re-emergence of the infection. In fact, in countries in which more than 70% of the population had been vaccinated, such as Singapore or United Kingdom, rebound in the numbers of newly infected cases was observed. Therefore, in addition to the vaccination, to comprehend the prevalence of the infected cases in an accurate and timely manner and make clear how the community and nosocomial transmission of the virus could take place, would be essential for the prevention of the resurgence.

In Japan, the cumulative number of the infected cases and that of deaths by COVID-19 as of March 2022 are approximately 5.6 million and 26,000, respectively and the incidence rate per 100,000 people is 4,436 [2], which is comparatively low when compared with countries in Europe, for example. When a frequency of contacting with patients with potential COVID-19 is considered, however, the risk of infection for healthcare workers (HCW’s) in a hospital in Japan that accommodate COVID-19 patients could be comparable as that of HCW’s in countries with the higher incidence rate.

Tokyo Shinagawa Hospital is a medium-sized hospital in Tokyo, Japan with 300 beds that has been accommodating and treating COVID-19 patients since March 2020. In this study, we investigated the prevalence of SARS-CoV-2 infection among HCW’s working in this hospital with PCR and two serological tests to address questions of, 1) whether the risk of infection is dependent on the frequency of contacting with patients with potential COVID-19, 2) how variable the positive rates would be by the detection methods, and 3) how much percentage of HCW’s is infected with SARS-CoV-2 without recognition, or silently infected.

## Materials and methods

### Study design

This study was conducted at Tokyo Shinagawa Hospital. The study protocol conformed to the ethical guidelines of the 1975 Declaration of Helsinki and was approved by the Ethics Committee at Tokyo Shinagawa Hospital (approval no. 20-A-34) prior to the start of the study. 685 HCW’s at Tokyo Shinagawa Hospital were included in this study after obtaining written informed consent. Sera were obtained from the subjects for the test of IgG against S protein’s receptor binding domain (IgG (RBD)) and IgG against nucleocapsid protein (IgG (N)) of SARS-CoV-2. None of them had been vaccinated with COVID-19 vaccine prior to the specimen collection. Subject information including age, sex, height, weight, job category in the hospital, history of SARS-CoV-2 infection diagnosed by PCR, and co-morbidity were obtained and anonymized before analyses.

Tokyo Shinagawa Hospital started accommodating COVID-19 patients in March 2020. All the HCW’s were provided with full personal protective equipment (PPE). Especially, all frontline HCW’s wore long-sleeved gowns, N95 respirators, gloves, goggles or face shields when treating patients. SARS-CoV-2 testing by PCR was promptly carried out for HCW’s who showed symptoms suggestive of COVID-19 including fever, dry cough, respiratory distress, loss of smell, or dysgeusia.

### Clinical and laboratory tests

Sera from the subjects were drawn during March and April 2021 and were tested with IgG (RBD) and IgG (N) assays. The titer of IgG (RBD) was measured by an ARCHITECT SARS-CoV-2 IgG II Quant assay on Architect i2000 CS5100 (Abbott Laboratories, Abbott Park, IL, USA) and that of IgG (N) was measured by an ARCHITECT SARS-CoV-2 IgG assay on Architect i2000 CS5100 (Abbott Laboratories, Abbott Park, IL, USA). According to the package insert of IgG (RBD) assay, the cut-off index is 50.0 AU/mL and lowest concentration at which CV% is within 20% is 7.8 AU/mL. Narasimhan et al. reported that the specificity of the IgG (RBD) assay was 100% while sensitivity of the IgG (RBD) exceeded 96% after 15 days from symptom onset by applying the cut-off of 50.0 AU/mL [5]. According to the package insert of IgG (N) assay, the cut-off index is 1.4 S/C and CV% at a mean index of 0.04 S/C of 50 negative controls is 5.9%. According to a report by Bryan et al., sensitivity and specificity of the IgG (N) assay assessed with series of specimens from 125 patients were both 100% after 17 days from symptom onset with the cut-off of 1.4 S/C [6]. In addition to this cut-off, we used 0.2 S/C as one of the candidate cut-offs for the long-term assessment of the history of SARS-CoV-2 infection by referring to the preceding literatures [7–10].

PCR was performed on QuantStudio™ 5 Real-Time PCR System (Thermo Fisher SCIENTIFIC, Waltham, MA, USA) which targets N1 and N2 regions of SARS-CoV-2. The PCR result was defined as positive when cycle threshold (Ct) was equal to or less than 40. Nasopharyngeal swabs for the PCR were collected by nurses who had ample experience of routine swab collections from fever outpatients and hospitalized patients.

### Statistical analysis

We used JMP 15.1.0 (SAS Institute Inc., Cary, NC, USA) for Pearson’s chi-square test in Table 3 in which antibody titers below the cut-off were categorized as “0” while those equal to or above the cut-off were categorized as “1”. P-value of <0.01 was considered significant.

## Results

### Characteristics of the study subjects

Baseline characteristics of the study subjects are presented in Table 1. Briefly, total subject number was 685 comprised of 199 male (29%) and 486 female HCW’s (71%). Medians of age and body mass index (BMI) were 31 years old and 21.5 kg/m^2^, respectively.

**Table 1:** Background characteristics of the study subjects.

		n	%	median
Subject number		685		
Male gender		199	29.1%	
Age				31.0
BMI	kg/m^2^			21.5
Chronic diseases				
	Hypertension	12	1.8%	
	Diabetes	2	0.3%	
	Dyslipidemia	7	1.0%	
	Bronchial asthma	21	3.1%	
	COPD	0	0.0%	
	Cardiovascular disease	2	0.3%	
	CKD	1	0.1%	
	Cancer	3	0.4%	

BMI, body mass index; COPD, chronic obstructive pulmonary disease; CKD, chronic kidney disease

### Positive rates of PCR, IgG (RBD) and IgG (N) by the job category

Overall positive rates and those by the job category of PCR, IgG (RBD) and IgG (N) were presented in Table 2. Regarding IgG (N), two cut-offs, 1.4 S/C and 0.2 S/C, were applied for the calculation of the positive rates and designated as IgG (N)_1.4_ and IgG (N)_0.2_, respectively. The overall positive rates by PCR, IgG (RBD), IgG (N)_1.4_, and IgG (N)_0.2_ were 3.5%, 9.5%, 6.1%, and 27.7%, respectively. In terms of absolute number of positive cases, nurses were at the highest by all the methods.

**Table 2:** Positive rates of PCR, IgG (RBD), IgG (N)_1.4_, and IgG (N)_0.2_ by job categories.

Role	n	%	PCR		IgG (RBD)		IgG (N)_1.4_		IgG (N)_0.2_	
			n	% pos.	Cut-off: 50 AU/mL		Cut-off: 1.4 S/C		Cut-off: 0.2 S/C	
					n	% pos.	n	% pos.	n	% pos.
Physician (respiratory)	2	0.3%	1	50.0%	2	100.0%	2	100.0%	2	100.0%
Physician (other)	48	7.0%	1	2.1%	2	4.2%	3	6.3%	13	27.1%
Dentist	1	0.1%	0	0.0%	0	0.0%	0	0.0%	0	0.0%
Nurse	267	39.0%	19	7.1%	45	16.9%	25	9.4%	84	31.5%
Nursing assistant	7	1.0%	0	0.0%	0	0.0%	0	0.0%	2	28.6%
Medical engineer	9	1.3%	1	11.1%	1	11.1%	1	11.1%	3	33.3%
Dental hygienist	6	0.9%	0	0.0%	0	0.0%	0	0.0%	1	16.7%
Midwife	2	0.3%	0	0.0%	0	0.0%	0	0.0%	0	0.0%
Physical therapist	125	18.2%	0	0.0%	4	3.2%	2	1.6%	27	21.6%
Radiologist	21	3.1%	0	0.0%	1	4.8%	0	0.0%	5	23.8%
Medical technologist	36	5.3%	0	0.0%	1	2.8%	1	2.8%	10	27.8%
Clinical trial	6	0.9%	0	0.0%	0	0.0%	0	0.0%	2	33.3%
Pharmacologist	14	2.0%	0	0.0%	0	0.0%	0	0.0%	4	28.6%
Dietitian	7	1.0%	0	0.0%	0	0.0%	0	0.0%	3	42.9%
Social worker	3	0.4%	1	33.3%	1	33.3%	1	33.3%	1	33.3%
Administration	131	19.1%	1	0.8%	8	6.1%	7	5.3%	33	25.2%
Overall	685	100.0%	24	3.5%	65	9.5%	42	6.1%	190	27.7%

### Positive rates between HCW’s in COVID-19 and non-COVID-19 ward

In Table 3, we compared positive rates in HCW’s who worked in COVID-19 ward and those who worked outside of COVID-19 ward by the four methods: PCR, IgG (RBD), IgG (N)_1.4_, and IgG (N)_0.2_. The positive rates in HCW’s in COVID-19 ward were significantly higher by Pearson’s chi-square test with all the methods.

**Table 3:** Positive rates of PCR, IgG (RBD), IgG (N)_1.4_, and IgG (N)_0.2_ in HCW’s working in COVID-19 or non-COVID-19 ward.

COVID-19 ward	n	%	PCR		IgG (RBD)		IgG (N)		IgG (N)	
					Cut-off: 50 AU/mL		Cut-off: 1.4 S/C		Cut-off: 0.2 S/C	
			n	% pos.	n	% pos.	n	% pos.	n	% pos.
Yes	57	8.3%	11	19.3%	23	40.4%	13	22.8%	33	57.9%
No	628	91.7%	13	2.1%	42	6.7%	29	4.6%	157	25.0%
p-value				<0.001		<0.001		<0.001		<0.001

### Incidence of SARS-CoV-2 infection along the timeline

**Fig 1:**
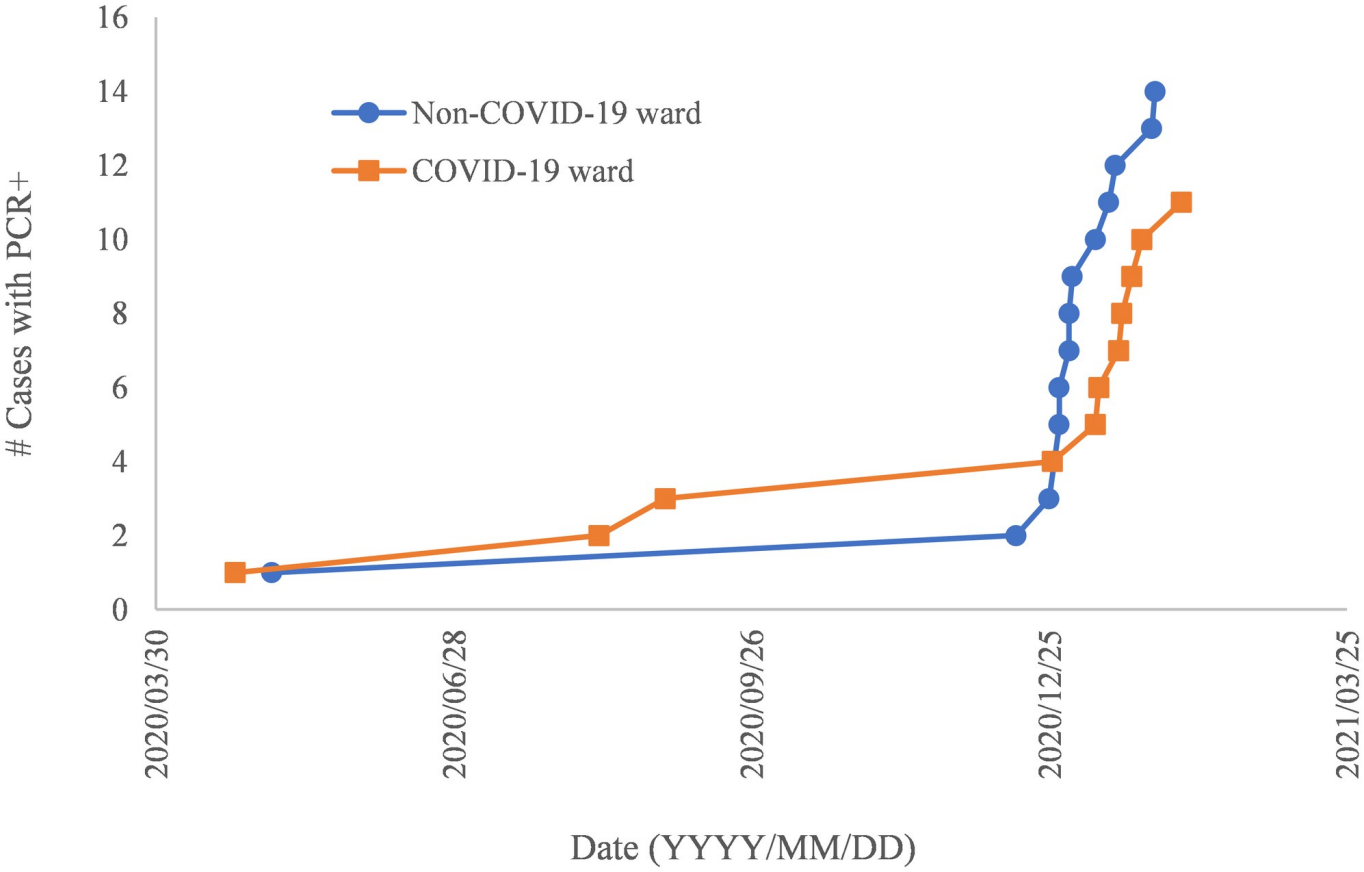
Accumulated number of cases with positive PCR results (y-axis) along with the confirmed timeline (x-axis). Blue circle, HCW’s in non-COVID-19 ward; orange square, HCW’s in COVID-19 ward.

We plotted the cumulative number of infected cases confirmed by PCR along the timeline of the period during April 2020 and February 2021 (Fig 1). At the end of December 2020, steep elevation in the number of positive cases was observed in HCW’s in non-COVID-19 ward, followed by HCW’s in COVID-19 ward by approximately two-week interval.

### Concordance and discordance of positivity among PCR, IgG (RBD), IgG (N)1.4, and IgG (N)0.2

**Fig 2:**
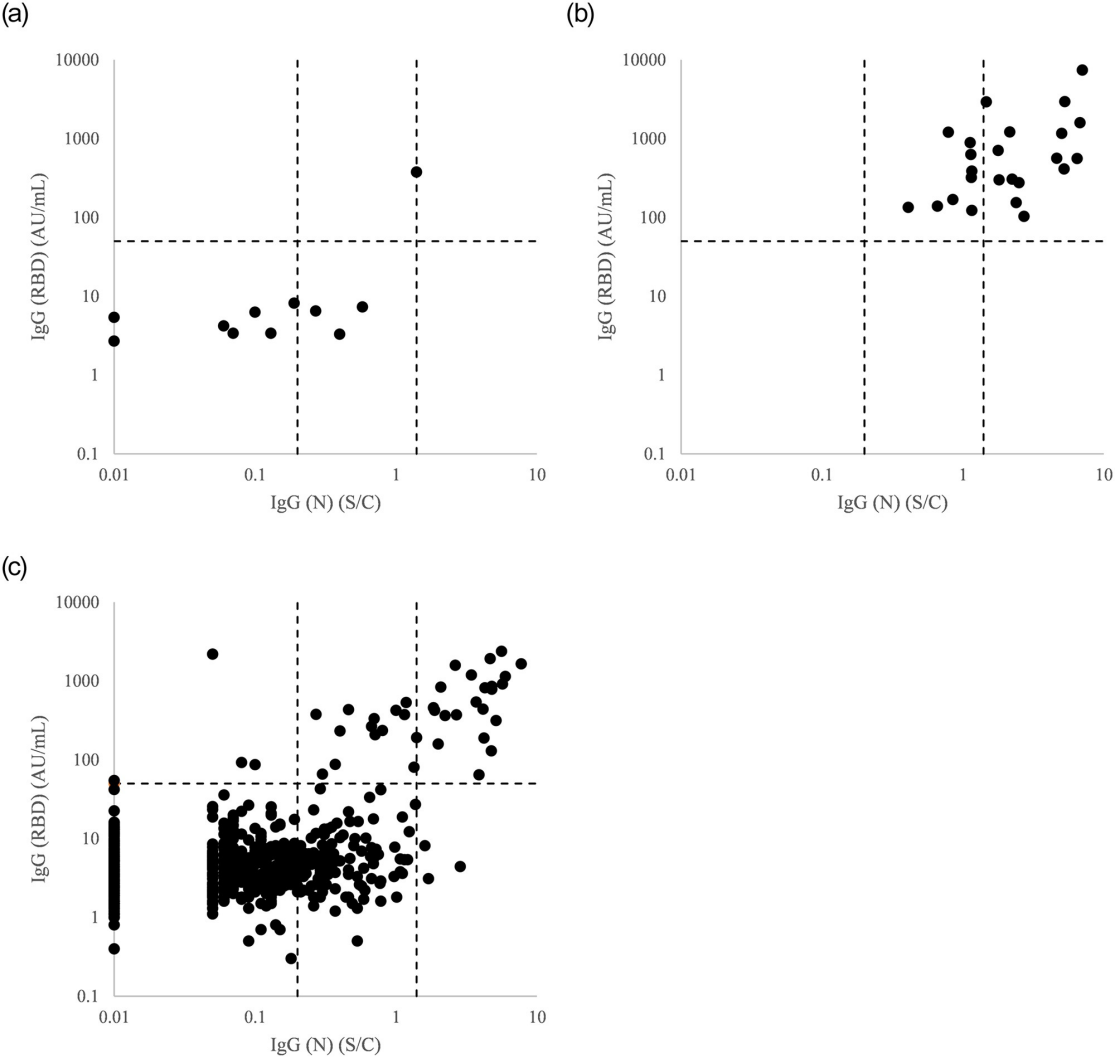
Distribution of IgG titers in healthcare workers in Tokyo Shinagawa Hospital. IgG (N) and IgG (RBD) titers are plotted on x- and y-axis, respectively. Dotted lines on x-axis are cut-offs of IgG (N) at 0.2 S/C and 1.4 S/C and that on y-axis is a cut-off of IgG (RBD) at 50 AU/mL. (a) Subjects with negative PCR; (b) Subjects with positive PCR; (c) Subjects without symptoms and had not been tested with PCR.

To examine the concordance and discordance among PCR, IgG (RBD), IgG (N)_1.4_, and IgG (N)_0.2_, we analyzed the distribution of IgG titers by plotting IgG (N) on the x- and IgG (RBD) on the y-axis with three subject groups: subjects with negative PCR results (Fig 2(a)), subjects with positive PCR results (Fig 2(b)), and subjects who had not been tested with PCR due to the lack of symptoms suggestive of SARS-CoV-2 infection (Fig 2(c)). The sensitivities, specificities, and concordances for IgG (RBD), IgG (N)_1.4_, and IgG (N)_0.2_ were shown in Table 4(a)–4(c). By the analysis in Fig 2(c) that examined 650 subjects who had not been tested with PCR, 40 subjects (6.2%), 26 subjects (4.0%), and 162 subjects (24.9%) were positive by IgG (RBD), IgG (N)_1.4_ and IgG (N)_0.2_, respectively.

**Table 4:** Sensitivity, specificity, and concordance of IgG (RBD), IgG (N)_1.4_, and IgG (N)_0.2_ against PCR.

(a)				
		IgG (RBD)		
		negative	positive	
PCR	negative	10	1	90.9%
	positive	0	24	100.0%
				97.1%
(b)				
		IgG (N)_1.4_		
		negative	positive	
PCR	negative	10	1	90.9%
	positive	9	15	62.5%
				71.4%
(c)				
		IgG (N)_0.2_		
		negative	positive	
PCR	negative	7	4	63.6%
	positive	0	24	100.0%
				88.6%

## Discussion

Among 685 HCW’s at Tokyo Shinagawa Hospital, the positive rates of PCR, IgG (RBD), IgG (N)_1.4_, and IgG (N)_0.2_ were 3.5%, 9.5%, 6.1%, and 27.7%, respectively (Table 2). The result that the positive rate of PCR was lower than those of serological tests is consistent with a result reported by Nakagama et al. [10]. This result not only suggests that positivity of PCR is an indication of ongoing infection while that of serological test is an indication of past as well as ongoing infection, but also indicates a possibility of a presence of cases with ongoing infection not detected by PCR but detected by serological tests. In fact, we reported the presence of cases suspected of SARS-CoV-2 infection due to symptoms and chest computed tomography (CT) findings characteristic with COVID-19 who never showed PCR positive even when the PCR was performed several times within five days from the onset, using IgG (RBD) and IgG (N) assays [9]. In this article, we discussed the transmissibility of the virus from patients infected with SARS-CoV-2 but without PCR positivity from five aspects: 1) unequal distribution of the viral load by the part of the body, 2) estimation of the probability of infectivity when given a negative PCR result, 3) dependance of transmissibility on the severity of the disease symptoms, 4) possibility of transmission via feces or urine, and 5) cases of infection cluster from patients with negative PCR. From that discussion, we concluded that the possibility of the viral transmission from those patients could not be excluded so that detection of the infection by serological tests could be useful to prevent further infection.

According to the survey conducted with 7,950 general population in Japan by the Ministry of Health, Labor and Welfare (MHLW) in June 2020, the positive rates of IgG (N)_1.4_ in Tokyo, Osaka and Miyagi were 0.20% (n = 1,971), 0.54% (n = 2,970) and 0.10% (n = 3,009), respectively [11]. In the second survey by MHLW conducted in December 2020, the positive rates of IgG (N)_1.4_ in Tokyo, Osaka and Miyagi were 1.09% (n = 3,399), 0.76% (n = 2,746) and 0.42% (n = 2,860), respectively [12]. Although the positive rates in all three areas were elevated in the second survey by several fold, the positive rate of IgG (N)_1.4_ in the HCW’s at Tokyo Shinagawa Hospital (6.1%) was higher than that in Tokyo in the second survey (1.09%) by more than five-fold. Herzberg et al. reported that positive rate of IgG (N)_1.4_ among HCW’s at a German secondary care hospital during March and June of 2020 was 1.52% [13]. Rodriguez et al. reported that positive rate of IgG (N)_1.4_ among HCW’s at a university hospital in Mallorca in Spain in April 2020 was 2.8% [14]. Lumley et al. reported that that positive rate of IgG (N)_1.4_ among HCW’s at Oxford University Hospitals was 9.8% by July 2020 [15]. Considering that the HCW’s in our study experienced four waves of the COVID-19 pandemic surges since March 2020 through April 2021, the positive rate of IgG (N)_1.4_ at Tokyo Shinagawa Hospital (6.1%) seems to be comparable to the ones at hospitals in those highly prevalent countries rather than the positive rate among the general population in Tokyo.

Regarding the job categories, nurses amounted 39% of all the HCW’s and were at the top in terms of absolute number of positive cases by all the four methods (Table 2). When compared with the second largest members at administrative division, nurses showed greatly higher positive rates (e.g., 7.1% vs. 0.8% by PCR), suggesting highly frequent contact with patients could be one of the sources of the infection. In addition to this, significantly higher positive rates were observed with all the four methods in HCW’s working in COVID-19 ward compared to the ones in HCW’s working outside of the COVID-19 ward (Table 3), which is also consistent with the report by Rodriguez et al. in which contact with patients infected with SARS-CoV-2 significantly increased the positive rate of the antibody among the HCW’s (p <0.001) [14]. It should be noted that the number of PCR positive cases abruptly increased at the end of December 2020 in HCW’s in non-COVID-19 ward and that it was followed by the increase in HCW’s in COVID-19 ward as shown in Fig 1. From this observation, it is inferred that not only the infection from patients infected with SARS-CoV-2, but also transmission among HCW’s could play significant role in the spread of the infection. In fact, Rodriguez et al. reported that not only contact with infected people but also contact with co-workers significantly increased the positive rate of the antibody among the HCW’s (p <0.001) [14].

We then examined the concordance and discordance among the methods. As shown in Fig 2(a), one subject was positive with IgG (RBD), IgG (N)_1.4_, and IgG (N)_0.2_ among 11 subjects with negative PCR results. Because all the three IgG tests showed positive with this one case, this subject may have been infected with SARS-CoV-2 but did not show positive by PCR due to the limited sensitivity of PCR [1, 9] or a silent infection after PCR was performed. If this subject is left out of the evaluation, the specificities become 100.0%, 100.0%, and 70.0% for IgG (RBD), IgG (N)_1.4_ and IgG (N)_0.2_, respectively. Regarding the three subjects who showed IgG (N) levels within the range of 0.2 and 1.4 S/C in Fig 2(a), IgG (RBD) were negative. For these subjects, two possibilities are considered: false positive with IgG (N)_0.2_, or true positive with IgG (N)_0.2_ despite negative results of PCR and IgG (RBD). The likelihood of the two possibilities depends on the validity of the cut-off 0.2 S/C. Narasimhan et al. suggested that the lower limit of IgG (N) among subjects with previous SARS-CoV-2 infection was 0.2 S/C [5]. In our previous study with subjects suggestive of SARS-CoV-2 infection but without PCR positivity, we derived 0.2 S/C as the cut-off of IgG (N) level by ROC analyses [9]. Eyre et al. reported that 15.5% of HCW’s with IgG (N) levels between 0.2 S/C and 1.4 S/C showed loss of smell and/or taste [8]. Therefore, within the range of 0.2 and 1.4 S/C, it may well be assumed that some, if not all, subjects are truly infected with SARS-CoV-2. On the other hand, the sensitivities for IgG (RBD), IgG (N)_1.4_, and IgG (N)_0.2_ with 24 subjects with positive PCR results were 100.0%, 62.5%, and 100.0%, respectively, which indicates that the sensitivity of IgG (N)_1.4_ may not be sufficiently high. When the sensitivity of IgG (RBD) and that of IgG (N)_0.2_ were to be compared further, the interpretation of the subject group with IgG (N)_0.2_ level between 0.2 and 1.4 S/C and with negative IgG (RBD) shown in lower middle in Fig 2(c) would be important. Among 148 subjects who showed IgG (N)_0.2_ level between 0.2 and 1.4 S/C, 22 subjects (14.9%) were with IgG (RBD) titers equal to or higher than 50 AU/mL, As mentioned earlier, 15.5% of HCW’s with IgG (N) levels between 0.2 S/C and 1.4 S/C showed loss of smell and/or taste [8]. If these subgroups coincide with each other, the rest of the 126 subjects (85.1%) who showed IgG (RBD) levels lower than 50 AU/mL may have been false positive for IgG (N)_0.2_. Moreover, Elslande et al. reported that half-life of IgG (RBD) titer was more than two-fold than that of IgG (N) titer and that IgG (RBD) titer was still positive in 92.4% of the subjects with previous SARS-CoV-2 infection at 7–10 months [16]. Therefore, it is more probable to assume that the 22 subjects with IgG (N) levels within the range of 0.2 to 1.4 S/C and with IgG (RBD) levels equal to or higher than 50 AU/mL were infected with SARS-CoV-2 some while ago so that the IgG (N) titers fell below 1.4 S/C. On the other hand, regarding the 126 subjects with IgG (RBD) levels lower than 50 AU/mL, it is less probable to assume that IgG (RBD) levels became below the cut-off faster than IgG (N) level against the report by Elslande et al. mentioned earlier [16]. One of the possibilities left for the true positiveness within the range of IgG (N) level of 0.2 to 1.4 S/C while IgG (RBD) is below the cut-off could be the difference in immunogenicity with nucleocapsid protein and S protein’s receptor binding domain: Burbelo et al. reported that antibody to the nucleocapsid protein of SARS-CoV-2 is more sensitive than spike protein antibody for detecting early infection [17]. Therefore, it may well be assumed that true and false positives of SARS-CoV-2 infection co-exist when IgG (N) is within the range of 0.2 to 1.4 S/C, so that IgG (N)_1.4_ could underestimate the long-term prevalence while IgG (N)_0.2_ could overestimate that.

Cao et al. reported that negative PCR results in COVID-19 patients could lead to increased risk of infection through the examination of cluster cases and pointed out that serological detection for SARS-CoV-2 infection would be an important tool in assisting the diagnosis of COVID-19 [18]. As shown in Table 2, the overall positive rate of PCR (3.5%) was the lowest when compared with the three serological tests. This is because the sensitivity of PCR was limited due to factors such as variable viral load or narrow window period for the detection [1], as well as that PCR test was limited to HCW’s who showed symptoms suggestive of SARS-CoV-2 infection because of its labor intensity and low throughput turnaround compared with other assays such as serological assays on an automated instrument. From the considerations on the results in Fig 2 mentioned above, IgG (RBD) is considered the most reliable method for detecting the ongoing SARS-CoV-2 infection in combination with PCR. Along with this line, the difference between the overall positive rate of IgG (RBD) (9.5%) and that of PCR (3.5%), which is 6%, could be considered the percentage of silent infection among the HCW’s. When we looked at the HCW’s working in COVID-19 ward, the positive rate of IgG (RBD) amounted to 40.4% while that of PCR was 19.3%, so that this difference (21.1%) could be considered the rate of silent infection in this subgroup. Johansson et al. reported that persons with SARS-CoV-2 infection who did not show any symptoms may account for approximately 24% of all transmission [19]. Cao et al., Rivett et al., and Zhao et al. also pointed out that asymptomatic infection among HCW’s could play substantial role in transmitting the virus [18, 20, 21]. For the prevention of nosocomial infection, HCW’s need to take precautions not only to transmission from patients infected with SARS-CoV-2 but also to transmission from co-workers with the silent infection. In this sense, serological test could serve as the indicator to detect the silent infection.

The difficulty with this, however, is that IgG (RBD) level is affected by vaccination with COVID-19 vaccine that targets spike protein of SARS-CoV-2 as the receptor binding domain is located within S1 subunit of spike protein in SARS-CoV-2. Because of this, use of IgG (RBD) test would need to be devised when the subjects have gone through the vaccination. For example, to monitor the potential break-through infection by IgG (RBD), the IgG (RBD) levels need to be periodically measured to obtain decaying comparator data. For the better prevention of nosocomial infection, earlier detection would be more advantageous. One way could be the combination assay with IgM and IgG. Another way could be the selection of target protein that elicits the immune response earlier than nucleocapsid protein or RBD: Liao et al. reported a possibility that detection of anti-SARS-CoV-2-S2 IgG was more sensitive than anti-RBD IgG in identifying asymptomatic COVID-19 patients [22], for example. The application of the early biomarker to the clinical practice, however, remains to be explored.

For the study of short- and long-term prevalence, on the other hand, IgG (N) has an advantage over IgG (RBD) because IgG (N) level is not affected by the vaccine that targets S protein. With the possibility of the presence of true positives within the range of 0.2 to 1.4 S/C of IgG (N) level as discussed earlier, IgG (N)_0.2_ could be the most reliable indicator among the three serological tests when all the possible infections including ongoing and past infections are to be detected.

In this study, 6.0% of overall HCW’s and 21.1% of HCW’s at COVID-19 ward were estimated to have been infected with SARS-CoV-2 without recognition, or silently infected, by the difference between IgG (RBD) and PCR. By detecting this silent infection in an accurate and timely manner, we would be able to minimize the occurrence of nosocomial infection cluster in the future.

## Conclusions

For the prevention of nosocomial infection of SARS-CoV-2, identification of silent infection is essential. For the detection of ongoing infection, periodical screening with IgG (RBD) in addition to PCR would be an effective measure. For the surveillance of morbidity in the population, IgG (N)_0.2_ could be the most reliable indicator among the three serological tests.
